# Two‐ Versus Four‐Narrow‐Implant‐Retained Dentures With Immediate‐Loaded Anterior Implants: 9 Years Randomized Clinical Trial

**DOI:** 10.1111/cid.70033

**Published:** 2025-05-09

**Authors:** Jana Kostunov, Nikolaos Nikitas Giannakopoulos, Peter Rammelsberg, Stefanie Kappel

**Affiliations:** ^1^ Department of Prosthodontics University of Heidelberg Heidelberg Germany; ^2^ Department of Prosthodontics University of Würzburg Würzburg Germany; ^3^ Department of Prosthodontics University of Athens Athens Greece

**Keywords:** dental implants, edentulous mandible, immediate loading, implant‐supported denture, late loading, Locator attachments, survival

## Abstract

**Introduction:**

In many clinical cases, anatomical conditions such as an atrophied alveolar ridge do not allow insertion of normal diameter implants. At this point, narrow dental implants may provide an effective option to avoid complex surgical treatments. The aim of this trial was to evaluate long‐term survival, prosthetic and biological complications, and OHRQoL for two‐piece narrow dental implants supporting overdentures with Locator‐attachments.

**Methods:**

Twenty‐five patients with edentulous mandibles received four narrow dental implants and retained dentures with Locator attachments. Following successful implantation, immediate loading of anterior implants with Locator attachments was performed, while posterior implants underwent a conventional healing procedure. Follow‐up examinations included documentation of implant and denture‐related complications, modified gingiva index, modified plaque index, OHIP‐G, and radiographic measurements of bone loss.

**Results:**

Up to 9 years after restoration, 18 patients with 72 implants were available for the follow‐up. During the observation period, one implant was lost in the immediate loading group. Implant survival was 98% and 100%. The outcomes of mPI and mGI for the 18 patients hardly differed between the groups. During the observation period, 136 prosthetic complications occurred. Scores for the different OHIP‐G domains at the last follow‐ups were stable over the years.

**Conclusions:**

Throughout an extended period of observation, we found stable implant survival rates, good maintenance of oral hygiene, and manageable prosthetic complications. The OHRQoL was satisfying over the years, suggesting that this treatment option could be favorable for older individuals.

**Trial Registration:**

https://drks.de/search/en/trial/DRKS00005497

## 1 Introduction

Edentulism is the complete absence of teeth that increases with age and is considered a disability by the World Health Organization [1]. This loss of teeth negatively affects chewing [2], which is essential for preparing the food bolus for swallowing and subsequent digestion [3]. Edentulism also degrades orofacial tissues, leading to a poor oral‐health‐related quality of life (OHRQoL) [4, 5, 6] and has been associated with higher mortality and morbidity [7].

The standard treatment for edentulism is a complete denture. These dentures significantly improve OHRQoL, particularly when supported by implants [8, 9]. However, normal‐diameter implants cannot be fitted in individuals with an atrophied alveolar ridge. Although invasive surgery can be performed to augment the ridge, these complex surgical interventions can be avoided by using narrow dental implants (3.0–3.5 mm) [10, 11, 12]. Some studies report high survival rates and improved oral health‐related quality of life (OHRQoL) with narrow implants, especially in elderly or medically compromised patients. For instance, Grandi et al. found that narrow implants (2.75 and 3.25 mm) in the posterior mandible demonstrated promising one‐year survival rates, highlighting their potential in scenarios where bone availability is limited [10]. The effect of loading time on the survival of narrow dental implants has been investigated. Many authors have shown adequate survival rates following immediate loading of narrow dental implants [13, 14, 15]. However, despite these early positive findings, there is substantial concern about the long‐term stability and load‐bearing capacity of narrow implants, especially under immediate loading conditions in edentulous patients. Clinical outcomes vary widely based on bone quality, implant design, and patient‐specific factors, such as occlusal forces and oral hygiene. For example, Bielemann et al. conducted a randomized clinical trial examining narrow implants under both immediate and conventional loading for mandibular overdentures and found that immediate loading increased peri‐implant inflammation and bone resorption, potentially reducing implant stability over time [16]. Additionally, Al‐Shibani et al. compared peri‐implant health between narrow and standard‐diameter implants over a 3‐year period in both diabetic and non‐diabetic patients. Their findings suggested that, while narrow implants can be successful, they may present a higher risk of peri‐implant mucositis and bone loss, especially in patients with compromised health, which can influence their suitability for high‐stress, edentulous applications. Other studies have shown good survival for narrow implants [17, 18].

The long‐term success of narrow implants also appears to be influenced by the number of implants used for denture retention. Some studies have shown that retaining dentures with just one implant is sufficient to increase OHRQoL [19], whereas others have recommended using two [20, 21] or even four or more [22] implants to improve implant survival. Kern et al. conducted a systematic review and found that overdentures supported by two narrow implants had higher implant loss rates compared to those supported by four implants. These results suggest that using a greater number of narrow implants may help distribute occlusal forces more evenly and enhance stability, although this approach may not be viable for all patients [23], whereas Mahgoli et al. found similar implant survival rates in individuals with mandibular overdentures supported by two or four implants. It seems that two implants are sufficient to retain mandibular dentures in the short term [24], but it is not clear if this is true for narrow implants with immediate loading. A study by Klein et al. comparing two and four narrow implant‐supported overdentures found that four‐implant configurations significantly improved load distribution and implant survival. This study concluded that, while two implants may provide sufficient initial retention, a four‐implant configuration in edentulous patients enhanced stability and reduced the risk of overload, especially with narrow implants [25]. Recent studies provide updated insights into the effectiveness of narrow‐diameter implants for mandibular overdentures. For example, a 2023 systematic review and meta‐analysis by Park et al. examined narrow versus regular‐diameter implants and found similar survival rates between both types. Narrow implants showed slightly improved outcomes in patient satisfaction and OHRQoL, making them a promising alternative in patients with limited bone volume [26]. However, bone loss and peri‐implant issues still raise concerns for immediate loading applications.

Attachments for implant‐retained dentures can increase the survival of implants and the satisfaction of patients. Unsplinted Locator attachments have become popular in recent years and have been described by various authors [15, 27] as a simple and affordable treatment option for the edentulous mandible [9]. These attachments function by connecting individual implants to a removable overdenture without linking the implants together with a rigid bar. Each locator attachment allows for a level of independent movement, providing flexibility for the denture during function. Unsplinted Locators have the benefit of a simple replacement or adjustment of individual abutments or clips if necessary, without affecting the entire prosthesis structure. Another advantage is the cost‐effectiveness. Unsplinted Locators tend to be less expensive, as they do not require complex fabrication or additional materials. Also, their placement and maintenance are generally less technique‐sensitive, allowing for a straightforward approach that is especially valuable for patients with reduced manual dexterity or when consistent follow‐up might be challenging. These attachments have been shown to improve masticatory performance and OHRQoL [28]. However, there are also some limitations. Unsplinted Locators do not evenly distribute masticatory forces across implants as effectively as splinted systems, which can lead to uneven stress on individual implants. This discrepancy may increase the risk of implant complications or wear on Locator attachments over time. There is also potential for increased wear. Independent movement can lead to accelerated wear on the attachments, necessitating more frequent replacement of components, such as retention clips or inserts.

In summary, while narrow implants can be a valuable solution in certain cases, their load‐bearing capacity, especially for immediately loaded overdentures, remains uncertain. This uncertainty underscores the need for further research to establish optimal patient selection and loading protocols to enhance outcomes for mandibular denture retention.

The aim of this trial was to evaluate the survival and prosthetic and biological complications of conventionally and immediately loaded two‐piece narrow dental implants in the interforaminal region of the narrow edge‐knife mandibular ridge supporting overdentures with Locator attachments. Furthermore, the OHRQoL of these patients should be observed over a 9‐year follow‐up period.

## 2 Material and Methods

### 2.1 Participants

**FIGURE 1:**
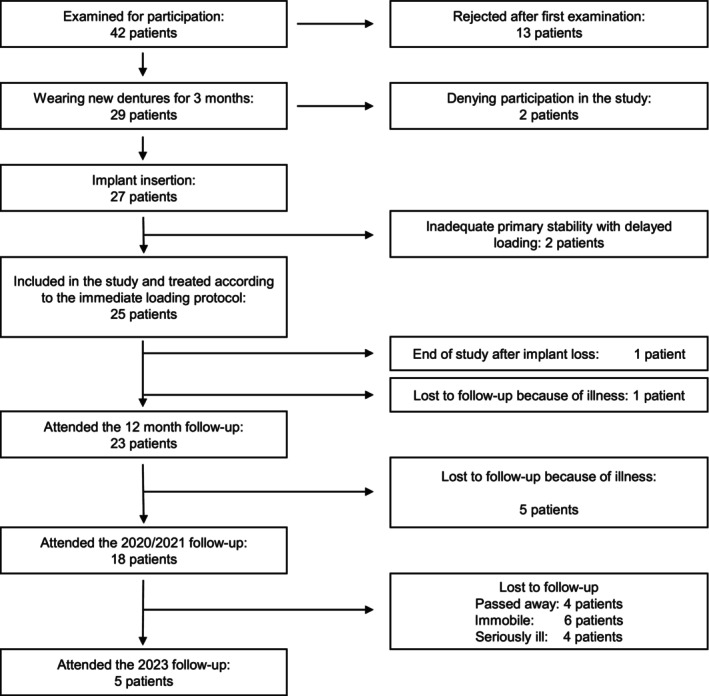
Flow diagram of the study participants.

Forty‐two individuals with edentulous mandibles (mean age, 69 years; 58% male) were initially screened for inclusion between 2012 and 2014 (Figure 1). Of these, 25 patients (mean age 67.35 years; 52% male) were included in the study and treated with the immediate‐loading protocol. The study was planned in accordance with the Consolidated Standards of Reporting Trials and approved by the ethics committee of the Medical Faculty of the University of Heidelberg (S‐161/2013). The study was registered in the German Registry for clinical trials (DRKS00005497). All participants were informed about the procedures, risks, and treatment, and they signed an informed consent form before inclusion in the study.

The 25 recruited patients had various types of maxillary prostheses: 18 had complete conventional dentures, 6 had removable partial dentures, and a few had fixed prostheses. Every patient was fitted with new complete mandibular dentures. Four two‐piece narrow, sandblasted, and etched conical and condensing implants were inserted in each patient (BEGO Mini Implant; BEGO Implant Systems GmbH and Co. KG, Bremen, Germany). All implants were 13 mm long, with a diameter of 3.1 mm. Following successful implantation, the anterior implants were immediately loaded with Locator analog attachments (diameter 3.5 mm; EasyCon, BEGO), while the posterior implants underwent conventional loading after an unloaded healing period of 3 months.

Participants were randomly assigned to one of two groups using sealed, non‐transparent, numbered envelopes that contained the randomized allocations for each patient. The randomization process was conducted by an individual not involved in the study at the Institute of Biometry and Informatics of the University of Heidelberg using randomization software. Envelopes were unveiled in a predetermined sequence 3 months following implant insertion, and patients were assigned to their respective treatment groups.

**FIGURE 2:**
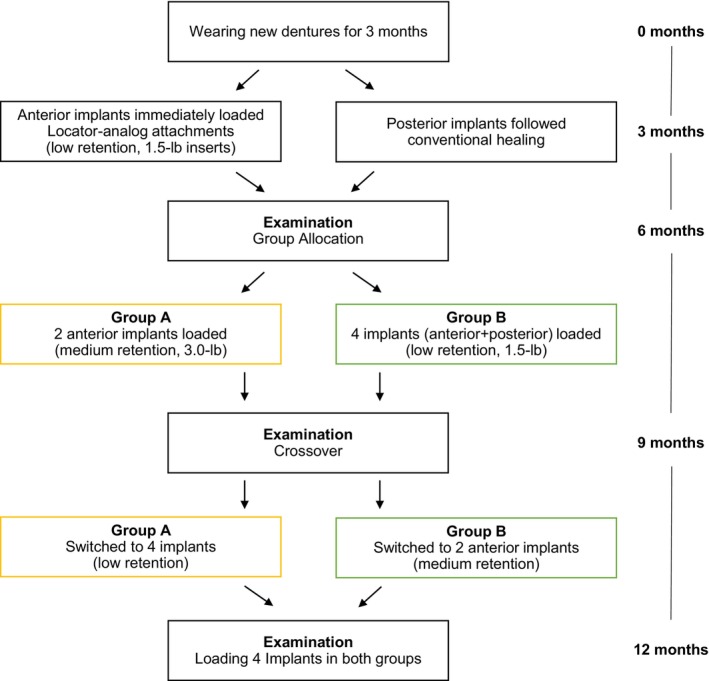
Allocation process chart.

Patients in the first group (Group A) were fitted with two Locator analog attachments on the anterior implants (medium retention) (3.0‐lb inserts), and patients in the second group (Group B) were fitted with four Locator analog attachments with the same level of retention as for immediate loading (low retention). According to the crossover design, patients were allocated to the other group after 3 months. This meant that, at 9 months after implant placement, each patient had been fitted with overdentures retained by four and two implants, loaded with Locator analog attachments (Figure 2).

We reported the 1‐year surgical outcomes in a previous publication [29]. Further follow‐up examinations were performed in 2021 and 2023, during which the same clinical assessments of both implants and dentures were performed, as described in our original study. Two examiners (S.K. and J.K.) contacted each patient either by telephone or by written correspondence to urge them to participate in the follow‐up examination at the clinic. Patients who could not be reached or who had died were recorded as dropping out of the study at their last clinical appointment.

It is essential to understand how implant‐retained mandibular dentures affect a patient's overall oral health, as this has a direct impact on their long‐term satisfaction and quality of life. This section examines the effects of these prosthetic solutions on oral hygiene, tissue health, and outcomes reported by patients. Oral health was evaluated using the modified gingiva index (mGI) and the modified plaque index (mPI), both of which have a scale of 0–3 [30]. Measurements were taken from the mesial, buccal, distal, and lingual faces of each implant. Next, peri‐implant mucositis or peri‐implantitis was screened according to the criteria defined by Berglundh et al. (2018). Mucositis was characterized by the presence of bleeding on gentle probing and the absence of bone loss beyond the crestal bone level. Peri‐implantitis was identified as bleeding on probing, increased probing depth, and the presence of bone loss [31].

Implant success after 9 years was determined using radiographic examinations, according to the Albrektsson criteria. In accordance with these criteria, an implant was defined as successful if marginal bone loss was limited to a maximum of 1 mm within the first year after insertion of the superstructure, and no greater than 0.2 mm in each subsequent year. For each patient, the distance between the implant apex and the last visible bone–implant contact was measured in 0.5‐mm steps at the distal and mesial faces of each implant on the panoramic X‐ray. This measurement was taken at the beginning of the study so that the results could be compared over time.

The OHRQoL of all patients was assessed using the German version of the oral health impact profile (OHIP‐G), which categorizes perceived OHRQoL into seven domains: (i) functional limitation, (ii) physical pain, (iii) psychological discomfort, (iv) physical disability, (v) psychological disability, (vi) social disability, and (vii) handicap. Each item is rated on a five‐point Likert scale, ranging from 0 (no discomfort) to 4 (high discomfort). The total score correlates inversely with the OHRQoL. Patients independently completed the self‐administered questionnaire in the waiting area, after which a study nurse (FD) reviewed the questionnaires for any missing data.

### 2.2 Statistical Procedures

All data were analyzed using IBM SPSS Statistics for Windows, Version 28.0. (IBM Corp. Released 2020. Armonk, NY, USA). Implant survival was estimated using Kaplan–Meier survival curves, which were generated individually for the immediate loading and late loading groups. Differences in survival rates between these groups were identified using the log‐rank test. Graphical representations illustrated prosthetic complications, denture aftercare, mGI, mPI, OHIP‐G score, and radiographic estimation of bone loss. The mGI, mPI, and OHIP scores were compared between the groups (immediate vs. late loading) using the Mann–Whitney U test. Questionnaire data were tabulated and assessed descriptively.

## 3 Results

Follow‐up examinations were performed until 2020/2021, during which a total of 18 patients with 72 implants still could participate. These patients had a mean age of 72.5 years (range, 64.9–85.8 years), and 50% were male (Table 1).

**TABLE 1:** Baseline characteristics of the 18 patients attending the 2021 recall.

Characteristics	Level	Number of patients
Age	< 60 years	0
	60–69 years	6
	70+ years	12
Gender	Female	9
	Male	9
Smoking	No	15
	< 10/day	1
	10–20/day	2
	> 20/day	0
Diabetes	No	11
	Type 1	0
	Type 2	7
Maxillary restoration	Complete denture	13
	Removable partial denture	4
	Fixed dental prosthesis	1

**FIGURE 3:**
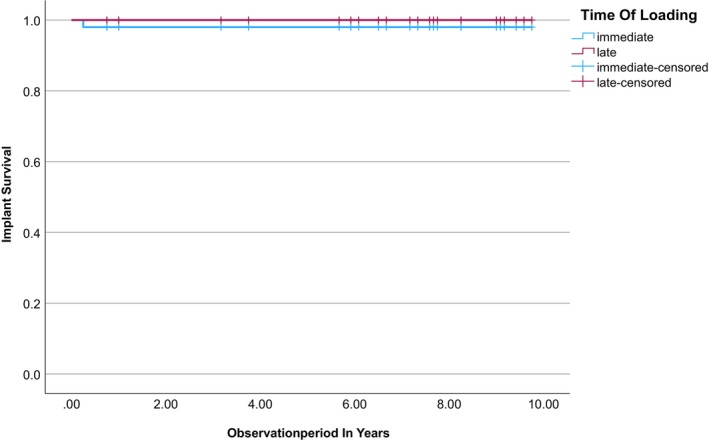
Kaplan–Meier curves for implant survival in the immediate and late loading groups.

During the entire observation period, one patient lost one implant in the immediate load group. According to the inclusion criteria, implant loss resulted in the end of study for this patient. Furthermore, six patients were lost to follow‐up because of serious illness. The implant survival rates were 98% (immediate load) and 100% (late load, Figure 3), and log‐rank tests revealed no statistically significant differences in implant survival between the two groups (*p* = 0.317).

We performed follow‐up examinations again in 2023, up to 9 years after the original restoration. All 19 patients were contacted again, but only five patients with a total of 20 implants could still participate in these examinations because of the following reasons: four patients passed away, six became immobile and could not visit the clinic, and four became seriously ill. The average age of these remaining five patients was 73.2 years (range, 68–79 years), and 40% were male (Table 2).

**TABLE 2:** Baseline characteristics of the five patients attending the 2023 recall.

Characteristics	Level	Number of patients
Age	< 60 years	0
	60–69 years	1
	70+ years	4
Gender	Female	3
	Male	2
Smoking	No	5
	< 10/day	0
	10–20/day	0
	> 20/day	0
Diabetes	No	3
	Type 1	0
	Type 2	2
Maxillary restoration	Complete denture	4
	Removable partial denture	1
	Fixed dental prosthesis	0

Mucositis and/or periimplantitis were evident in four implants in one patient and were treated accordingly. Marginal bone loss ranged from 0 to 3 mm (Tables 3 and 4). We calculated the overall implant success rate 9 years after the original restoration, according to the Albrektsson criteria (which consider bone resorption) and the criteria outlined by Berglundh et al. (2018) (which consider bone resorption and gingival condition), and found that the overall implant success rate decreased to 96% in the late loading group and to 94% in the immediate loading group. The mPI and mGI scores were not different between groups at the 2020/2021 follow‐up (18 patients, Table 5) or at the 2023 follow‐up (five patients, Table 6).

**TABLE 3:** Marginal bone loss (mm) measured at the mesial and distal faces for all implants at the 2020/2021 recall.

Marginal bone loss in (mm)	Number of implants (measurements at mesial faces)		Number of implants (measurements at distal faces)	
	Immediate	Late	Immediate	Late
0	15	20	16	18
0.5	1	1	3	2
1.0	7	7	8	7
1.5	5	2	2	2
2.0	2	0	1	0
2.5	0	0	0	0
3.0	0	0	0	1
3.5	0	0	0	0
4.0	0	0	0	0
4.5	0	0	0	0
No X‐ray	12			

**TABLE 4:** Marginal bone loss in mm measured at the mesial and distal faces for all implants at the 2023 recall.

Marginal bone loss in (mm)	Number of implants (measurements at mesial faces)		Number of implants (measurements at distal faces)	
	Immediate	Late	Immediate	Late
0	2	3	5	3
0.5	4	1	1	2
1.0	4	5	2	3
1.5	0	0	0	1
2.0	0	1	1	1
2.5	0	0	0	0
3.0	0	0	1	0
3.5	0	0	0	0
4.0	0	0	0	0
4.5	0	0	0	0
No X‐ray	0			

**TABLE 5:** Modified plaque index (mPI) and gingiva index (mGI) of patients participating in the 2020/2021 recall (*N* = 18 patients).

Score on scale	Number of implants for mPI		Number of implants for mGI	
	Immediate	Late	Immediate	Late
0	17	19	22	22
1	13	14	11	10
2	6	3	2	3
3	0	0	1	1

**TABLE 6:** Modified plaque index (mPI) and gingiva index (mGI) of patients participating in the 2023 recall (*N* = 5 patients).

Score on scale	Number of implants for mPI		Number of implants for mGI	
	Immediate	Late	Immediate	Late
0	2	3	2	4
1	5	4	6	4
2	3	3	2	2
3	0	0	0	0

During the 9‐year study period, 136 prosthetic complications required treatment. Most interventions were for minor complications, such as changing one or all of the retention clips (Table 7), and nine Locator abutments were replaced because of wear. Notably, there was no loss of superstructure or need to remake dentures. However, one denture required adaptation after implant failure.

**TABLE 7:** Prosthetic complications of all patients during the observation period.

Type of complication	Immediate/late group
Change of locator abutment	9
Change of the clips	105
Re‐fixing of the retention clips	1
Relining of mandibular denture	12
Loosening of locator	1
Fracture of acrylic teeth	5
Micro crack in mandibular denture	3
Total	136
Patients without prosthetic complications	0

**FIGURE 4:**
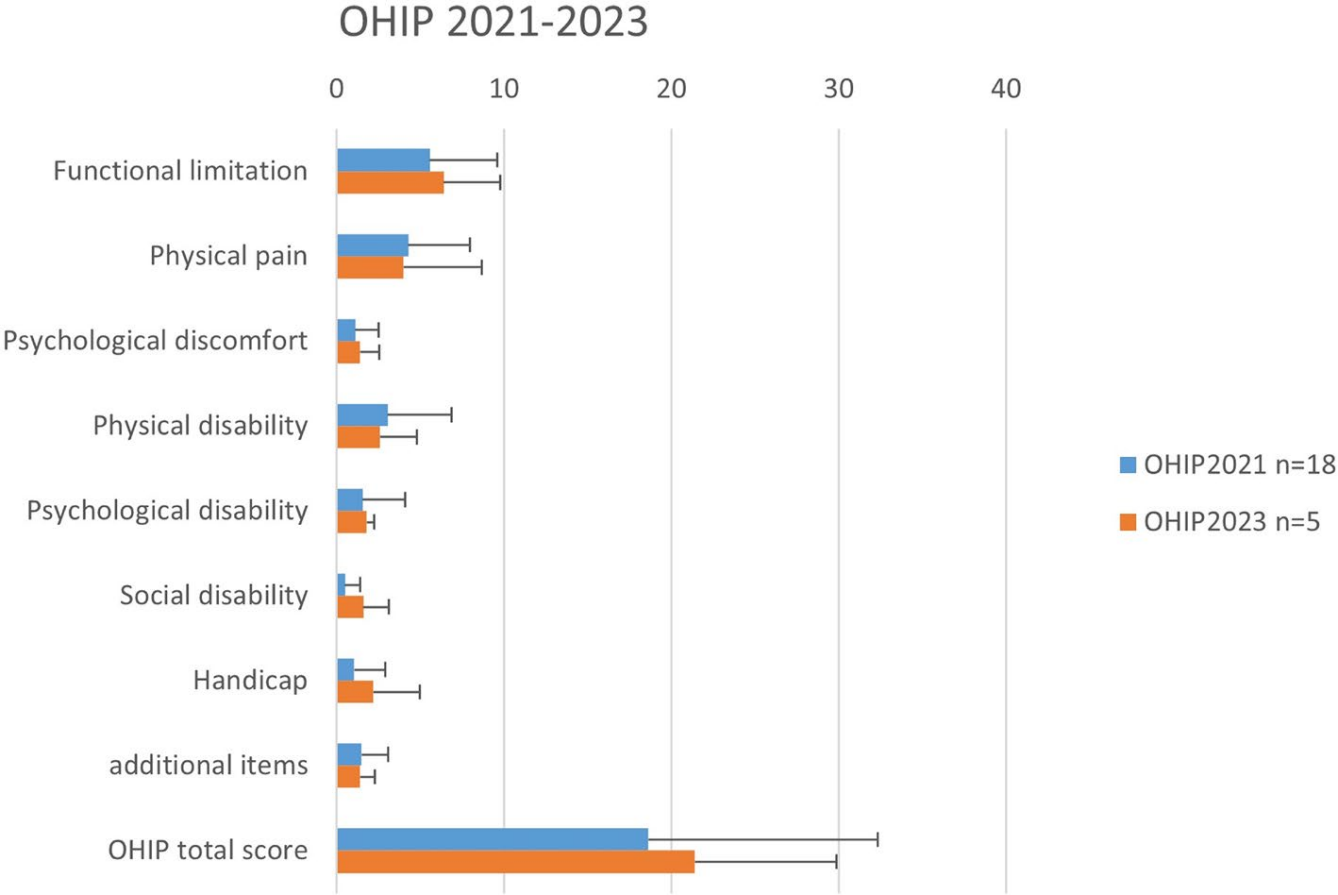
Scores for the different OHIP‐G domains at the 2021 and 2023 recall.

The mean (SD) total OHIP‐G score of the 18 patients at the 2020/2021 recall was 18.61 (13.72), and scores for the different OHIP‐G domains are presented in Figure 4. The mean (SD) total OHIP‐G score of the five patients at the 2023 recall was 21.4 (8.44). There were not enough patients at the last recall to compare the OHIP scores with inferential statistical tests. All patients who participated in the 2020/2021 and 2023 recalls said they would still recommend the treatment.

## 4 Discussion

This study assessed the outcomes of mandibular overdentures subjected to immediate and late loading over a 9‐year period. Although the effects of immediate and late loading on dentures have been extensively documented, the long‐term effects on dentures with Locator attachments remain scarce. In the present study, implant survival was 100% in the late loading group and 98% in the immediate loading group. We also showed an implant success rate of 96% after late loading and 94% after immediate loading.

Similar to our findings, Rignon et al. reported a 10‐year implant survival rate of 96.5% after immediate loading of dentures with ball attachments [32]. Another study reported a 5‐year implant survival rate of 100% after both immediate and late loading of implants with Locator attachments. However, this study only examined implants with a diameter of 4.0 mm [15]. Possebon et al. reported a 100% 3‐year survival rate of narrow dental implants after both immediate and late loading [33], and Salman et al. similarly reported a 100% 5‐year implant success rate in both groups [15]. A similar success rate was reported by Büttel et al., 2 years after restoration with immediately loaded standard implants [34]. Ates et al. demonstrated an 85.7% success rate for immediately loaded implants with Locator attachments after a 5‐year observation period [35].

Eighteen patients participated in our 7‐year follow‐up, while only five participated in the 9‐year recall because of increasing age. Recruiting older patients for long‐term observations is typically challenging because of death, illness, relocation to nursing homes, and other reasons, so it is very valuable to have this number of patients.

Prosthetic complications were prevalent during the study, but all were effectively addressed. The most severe intervention was the replacement of Locator abutments, while relatively less‐severe interventions included refixing retention clips, relining the dentures, and restoring micro‐cracks in the denture (Table 7). In agreement with our findings, changing retention clips was the most common complication in other studies [36]. Compared with alternative attachment systems, Prasad et al. found fewer complications with Locator attachments than with bar‐type attachments or magnetic‐retention systems [37].

The mPI and mGI scores were not different after immediate and late loading, and mucositis and peri‐implantitis were only found in one patient, a lower prevalence than that reported previously. Peri‐implant health is determined by bone resorption and gingival condition, according to a consensus report of 2017 [31]. Peri‐implant health was evaluated according to these updated criteria in the present study, and we observed the same outcomes as with the mGI and Albrektsson criteria. In a systematic review, the prevalence of peri‐implant mucositis ranged from 19% to 65%, with a weighted mean prevalence of 43% [38]. Elderly patients often have difficulty brushing their teeth properly; therefore, an unsplinted attachment may be preferable in these individuals to help them improve their oral hygiene.

The retention and stability of removable dentures decline over time in edentulous patients because of the gradual resorption of the mandible. It has been shown that overdentures retained by implants can enhance the OHRQoL of elderly edentulous patients. In this study, it was shown that retention with more than two implants can improve patients' quality of life. These findings align with those of other authors. For example, Kheur et al. found that overdentures retained by three implants resulted in better OHRQoL scores and higher patient satisfaction than overdentures retained by two implants or conventional complete dentures did [39]. Other authors have reported better OHRQoL in patients with implant‐retained overdentures than in those with conventional dentures. The OHRQoL is also significantly higher when four implants, rather than two, are used in Locator‐retained overdentures [40].

Although we could not use inferential statistics because the number of patients was too low at the final recall, we assume that the OHIP score was stable between the last two recalls. Moreover, the absolute difference between the OHIP values did not exceed the minimal clinically important difference of 14 points, as suggested by other authors [41]. These results are comparable with those of other authors, showing that OHIP values remain stable over time. For example, Mukilvannan et al. reported high OHIP scores and satisfaction over 8–10 years in edentulous patients treated with implant mandibular overdentures [42]. Other authors have shown that OHIP scores improved after restoration and stayed stable over 5 years [43].

A limitation of this study was the low number of patients available for follow‐up over the up to 9‐year observation period. However, despite this limitation, our findings show that immediate or late loading of overdentures retained by four mandibular implants using Locator attachments is a clinically feasible treatment option. The outcomes indicated a high implant success rate, stable clinical conditions, and manageable prosthetic complications.

## 5 Conclusions

We found high long‐term implant survival rates overall, with no significant differences noted between the anterior implants, which were immediately loaded, and the posterior implants, which underwent late loading. Oral hygiene was consistently maintained across the cohort, prosthetic complications were manageable, and OHRQoL remained stable over the years. Therefore, the use of reduced‐diameter implants with Locator attachments, immediately loaded or late loaded, appears to be a viable and suitable treatment option for older individuals. These findings may assist clinicians in designing effective dental treatment plans for edentulous patients.

## Author Contributions

Jana Kostunov acquired the data, analyzed and interpreted the data, wrote the original draft and reviewed and edited the paper. Nikolaos Nikitas Giannakopoulos analyzed and interpreted the data, reviewed and edited the paper, and administrated the project. Peter Rammelsberg supervised the project, reviewed and edited the paper, and curated the data. Stefanie Kappel conceived and designed the project, acquired the data, wrote the original draft, performed the methodology, and acquired resources.

## Ethics Statement

All procedures in this study involving human participants were performed in accordance with the ethical standards of the institutional committee and with the 1964 Declaration of Helsinki and its later amendments or comparable ethical standards.

## Consent

All patients gave written informed consent to participate before inclusion in the study.

## Conflicts of Interest

The authors declare no conflicts of interest.

## Supporting information


**Data S1.** CONSORT‐2010‐Checklist.

## Data Availability

The datasets generated and analyzed during the current study are available from the corresponding author on reasonable request.
